# CuO Modified by 7,7,8,8-Tetracyanoquinodimethane and Its Application to CO_2_ Separation

**DOI:** 10.3390/ijms232314583

**Published:** 2022-11-23

**Authors:** Juyeong Lee, Sangwook Kang

**Affiliations:** 1 Department of Chemical Engineering and Material Science, Sangmyung University, Seoul 03016, Republic of Korea; 2 Department of Chemistry and Energy Engineering, Sangmyung University, Seoul 03016, Republic of Korea

**Keywords:** 7,7,8,8-Tetracyanoquinodimethane (TCNQ), polyvinylpyrrolidone (PVP), CO_2_ separation, interaction, free volume

## Abstract

7,7,8,8-Tetracyanoquinomethane (TCNQ) was added to polyvinylpyrrolidone (PVP)/CuO composites to modify and prevent agglomeration of the particles, and thus the CuO particles were well dispersed to a small size, thereby increasing CO_2_ solubility and separation performance. When the separation performance of the PVP/CuO/TCNQ composite membrane was measured for CO_2_/N_2_ gases, a CO_2_ separation of about 174 was measured. This improvement in performance was attributed to the fact that TCNQ was applied to PVP and CuO to prevent agglomeration between particles with surface modification. Due to TCNQ, CuO could be dispersed to a small size in PVP; the bonds between chains in PVP weakened; the interaction between molecules weakened; and the free volume increased, as confirmed by FT-IR, TGA, and UV–Vis spectroscopy.

## 1. Introduction

As the world’s population grows and industry develops, the increase in the use of fossil fuels has led to a surge in CO_2_ emissions, which are responsible for global warming [1]. The increase in CO_2_ emissions due to fossil fuels affects the atmospheric heat balance [2]. An increase in carbon dioxide causes various environmental problems, such as affecting the thermal balance of the atmosphere. Global temperatures rise and glaciers decrease, affecting various areas throughout agriculture and ecosystems [3]. Therefore, in order to mitigate such environmental problems, the stabilization target of the concentration for greenhouse gases in the atmosphere was set at a level consistent with the need for sustainable development. In order to achieve CO_2_ emission reduction, focusing on collecting CO_2_ from fossil fuel combustion is so important [4]. Reducing CO_2_ emissions in the process of burning fossil fuels, which accounts for about 95% of global emissions, is attracting attention as an important issue [5,6]. Research on CO_2_ collection technology has been conducted as a method for reducing the amount of gases released into the atmosphere. It is attracting attention as a technology used to recover and select CO_2_ from large-scale emission sources [7,8,9]. Various methods have been proposed to capture CO_2_ emissions, such as absorption using various liquid solvents, separation using techniques such as membranes, adsorption using pressure or temperature [10], and CCS technology for capturing and storing carbon, which can reduce the amount of CO_2_ emitted into the atmosphere [11]. Among the CCS technologies, the method using membranes has the advantage of being inexpensive and having high functionality in various industrial applications. As the use of additional materials, such as the separation agent or phase change, is not required, it is possible to reduce costs and suggest various functions. In addition, membranes can be used both before and after combustion in the process, and have low weight and maintenance [12,13,14,15]. Membrane technology such as this is used in various fields such as gas and steam separation and fuel cells [16]. In addition, research on the advantages of such membranes is being actively conducted. For example, high performance for CO_2_ separation was reported when membranes were prepared using a polyethylene oxide-poly butylene terephthalate multiblock copolymer with a specific additive [17]. Recently, a new type of mixed matrix film (MM) was researched, integrating inorganic particles into a polymer matrix instead of a polymer film or an inorganic film as a filler. Research was conducted to improve existing limitations and disadvantages. The most important part of this study was the use of filler to control the gas separation characteristics of the membrane. Materials such as zeolite, carbon, and metal organic skeleton were used as fillers, which change the permeability and selectivity of CO_2_ [18]. Furthermore, mixed matrix membranes (MMMs) have been reported. For mixed matrix membranes manufactured with polymer and silica nanoparticles with gas-permeable nanospace, the CO_2_ permeability of the silica nanoparticles increased with the nanospace [19]. The limitation of this mixed matrix is that it has an abnormal interface shape, which weakens the gas separation performance and mechanical strength. In order to solve these disadvantages, a study applied amino-silane functionalized graphene oxide to a Pebax 1657 matrix. It reported that the amino group of GO improved CO_2_/N_2_ selectivity to 71.1 by assisting with the formation of a transport path along the polymer–pillar interface [20]. Moreover, BI-PEG-xPI was used as a cross-linking agent based on PEG for CO_2_ separation. Both an ionic group and a PEG were used as crosslinking agents to suppress plasticization and as a CO_2_ solubilizing group to improve CO_2_ selectivity. As a result, the CO_2_/N_2_ selectivity was improved to 31 [21]. In addition, researchers developed an optimal polymer matrix by mixing hydrophilic polymer PVP and PVA. The permeability of CO_2_ as a material to facilitate CO_2_ transport almost doubled [22]. The mixed matrix membrane produced using sulfonated poly(ether ether keton) (SPEEK) and amine-functionalized titanium submicropores increased the content of the membrane’s promoted transport sites due to PEI with a voluminous amine group. Thus, the gas permeability and selectivity were largely enhanced. The membrane had a CO_2_/N_2_ separation performance of 64 and a CO_2_ permeance of 1629 Barrer [23]. Research is being conducted on improving the permeance and selectivity and resolving disadvantages by manufacturing membranes in various ways.

Research on separating CO_2_ with a composite membrane is actively being conducted. For example, membranes including ZnO nanoparticles were prepared using an ionic liquid and used for CO_2_/N_2_ separation. This improved the overall performance by increasing the solubility of CO_2_ and acting as a barrier to N_2_ in the oxide layer of ZnO nanoparticles with 1-butyl-3-methyl imidazolium tetrafluoroborate (BMIMBF_4_) in the ionic liquid. This showed a separation performance of 101 GPU and selectivity of 42.1 [24]. Researchers used aniline as a membrane composed of other components. In this experiment, an additive containing benzene was used. Aniline was incorporated into the polymer membrane because it had both benzene rings and amino groups, and selectivity was improved by the effects of both gas barriers and CO_2_ carriers. Therefore, this membrane could be effectively used for CO_2_/N_2_ separation. The separation performance of this membrane was improved by about 80 times compared with that of a neat PVA membrane [25]. A composite CO_2_ separation membrane was produced using 1-aminopyridinium iodide (iodine salt) to improve the separation performance to CO_2_/N_2_ selectivity to 64.6 and permeance of CO_2_ gas to 22.6 GPU. This material promoted the transport of N_2_ molecules by cyclic compounds and the transport of CO_2_ molecules by amine groups [26]. Studies on CO_2_ separation membranes using ionic liquids BMIMBF_4_ and CdO nanoparticles have also been reported. The solubility of CO_2_ was increased by free ions present in the oxide layer of the CdO nanoparticle ionic liquid. As a result of significantly improving the separation performance using iodine salt, the CO_2_/N_2_ selectivity was 64.6, and CO_2_ transmittance was 22.6 GPU, indicating a performance twice that of the BMIM^+^BF_4_^−^/CdO composite without iodine salt [27]. A poly(ethylene oxide) (PEO)-based separation membrane was manufactured using 5-hydroxyisophthalic acid. These membranes improved the ideal selectivity of CO_2_ by reducing the transport of N_2_ through providing a barrier effect owing to the solubility of CO_2_ and the benzene ring. As the transmittance of N_2_ reduced, the selectivity of CO_2_ improved. In this study, CuO particles and 7,7,8,8-tetracyano quinodimethane (TCNQ) were utilized for their high separation performance and low-cost process based on polymer composite membranes.

## 2. Results and Discussion

### 2.1. Scanning Electron Microscope (SEM)

**Figure 1:**
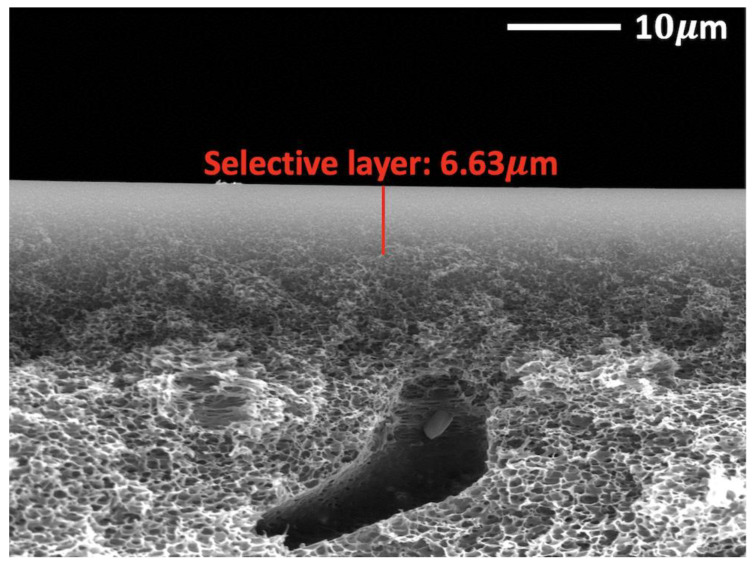
SEM image: PVP/CuO/TCNQ composite membrane coated onto polysulfone support.

Figure 1 shows the thickness of a film coated with PVP/CuO/TCNQ on porous polysulfone. The relatively pale part of the porous polysulfone (sponge-like structure) indicated the penetration of PVP/CuO/TCNQ composite into the support. The SEM image showed that the thickness of the selective layer was 6.63 μm.

### 2.2. Fourier Transform Infrared (FTIR)

**Figure 2:**
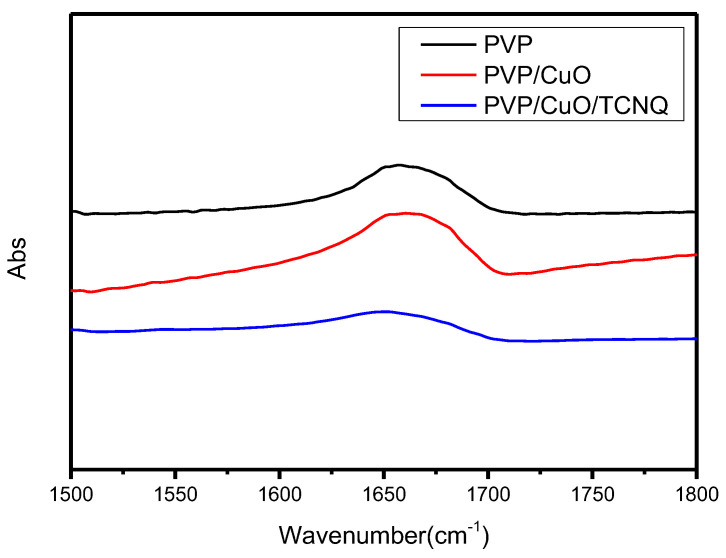
IR spectra of PVP, PVP/CuO, and PVP/CuO/TCNQ composites.

**Figure 3:**
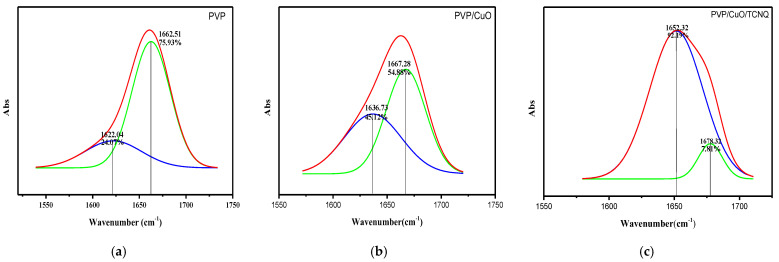
Deconvoluted IR spectra of (**a**) PVP, (**b**) PVP/CuO composite, and (**c**) PVP/CuO/TCNQ composite.

FT-IR was measured to determine the interaction between PVP, CuO, and TCNQ. According to Figure 2 and Figure 3, when CuO was added to PVP as a polymer and TCNQ was added, the absorption band at 1650 cm^−1^ occurred. In the case of neat PVP, a peak occurred at 1650 cm^−1^, representing the carbonyl groups, and the result of adding CuO at a ratio of 1:1 to PVP produced the absorption band seen in Figure 3b. In addition, Figure 3c shows when TCNQ was added at a 1:1:0.1 ratio with PVP/CuO/TCNQ molar ratio. When CuO and TCNQ were applied, deconvolution was performed to determine the change in the carbonyl groups, and, as a result, it was observed that the peak of 1650 cm^−1^ moved to a higher wavenumber compared with those of neat PVP and PVP/CuO.

TCNQ was sequentially added. These results showed that the carbonyl bond was strengthened by applying CuO and TCNQ, and the hydrogen bond between macromolecular chains weakened. In addition, it can be assumed that the free volume was relatively increased because the intermolecular interaction between chains was weakened.

### 2.3. TGA

**Figure 4:**
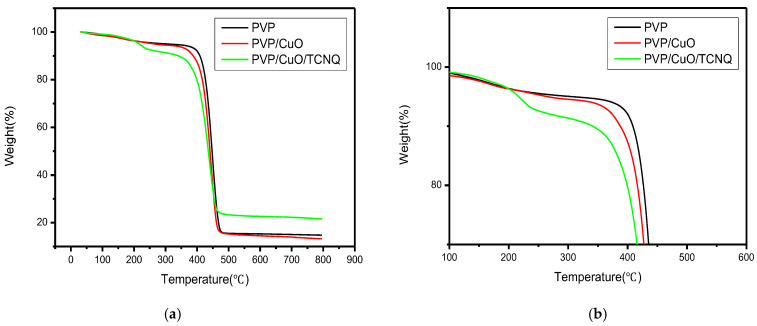
(**a**) TGA curves of PVP/CuO and PVP/CuO/TCNQ composites, and (**b**) enlarged image of (**a**) from 100 to 600 °C.

**Figure 5:**
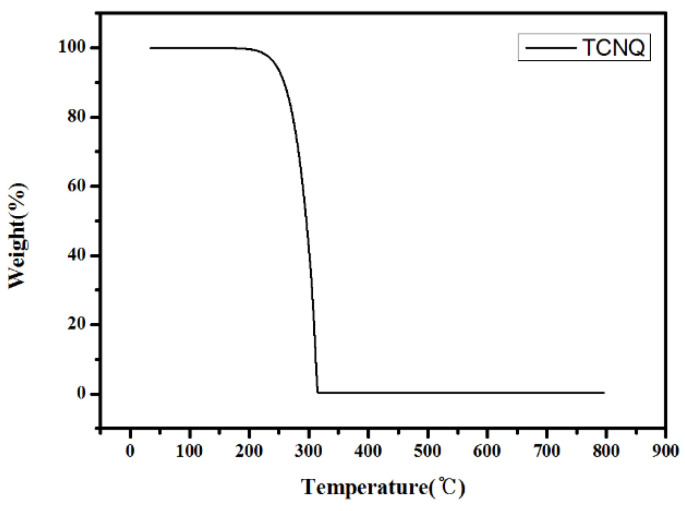
TGA curve of TCNQ from 100 to 600 °C.

The thermal stability of the PVP, PVP/CuO, and PVP/CuO/TCNQ composite was measured using thermogravimetric analysis. The interaction between PVP, CuO, and TCNQ was investigated. As shown in Figure 4a,b and Figure 5, PVP showed weight reduction at about 400 °C. In the case of the PVP/CuO composite, it could be observed that weight reduction occurred at about 370 °C, which is at a lower temperature than that of neat PVP. In the case of the PVP/CuO/TCNQ composite, the first weight reduction occurred at about 230 °C, and the second weight reduction occurred at about 350 °C. For the TGA curve of pure TCNQ, it could be observed that weight loss occurred at a temperature of about 250 °C. When only CuO was applied to PVP, the overall weight reduction pattern was similar even though weight reduction occurred at a lower temperature than that of neat PVP. However, when TCNQ was applied, the overall weight loss occurred at a low temperature, which meant that the bonds between chains became weakened. Furthermore, it indicated that the intermolecular interaction decreased, resulting in the increase in free volume.

### 2.4. UV–Vis Spectroscopy

**Figure 6:**
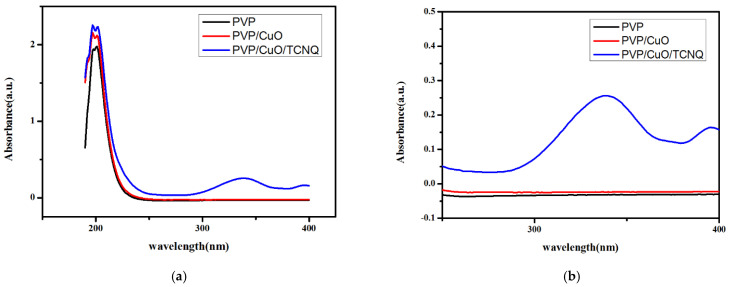
(**a**) UV–Vis spectrum of PVP, PVP/CuO, and PVP/CuO/TCNQ composites, and (**b**) enlarged image of (**a**).

**Figure 7:**
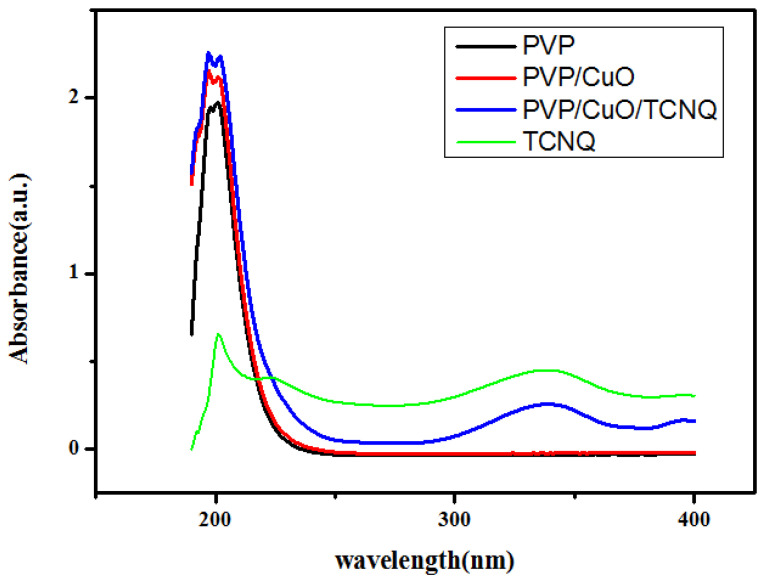
UV–Vis spectrum of PVP, TCNQ, PVP/CuO, and PVP/CuO/TCNQ composite.

The porosity of the CA film was measured using the mercury intrusion method. Figure 6 and Figure 7 show the UV–Vis spectroscopy for each solution of the PVP, PVP/CuO, and PVP/CuO/TCNQ composites. In the case of a solution of PVP and PVP/CuO, oscillation was not observed at a specific wavelength, whereas when TCNQ was applied, oscillation was exhibited at a range of 300 to 350 nm. It can be seen in Figure 7 that the change was caused by TCNQ.

This observation can be explained by the fact that before TCNQ was added, the particle size of CuO was relatively large, and thus the vibrating wavelength did not appear on UV–Vis. On the other hand, the particle was maintained in a small size and well dispersed due to the interaction of TCNQ with the surface of CuO, and thus the oscillation was observed in a specific wavelength.

### 2.5. Separation Test

**Figure 8:**
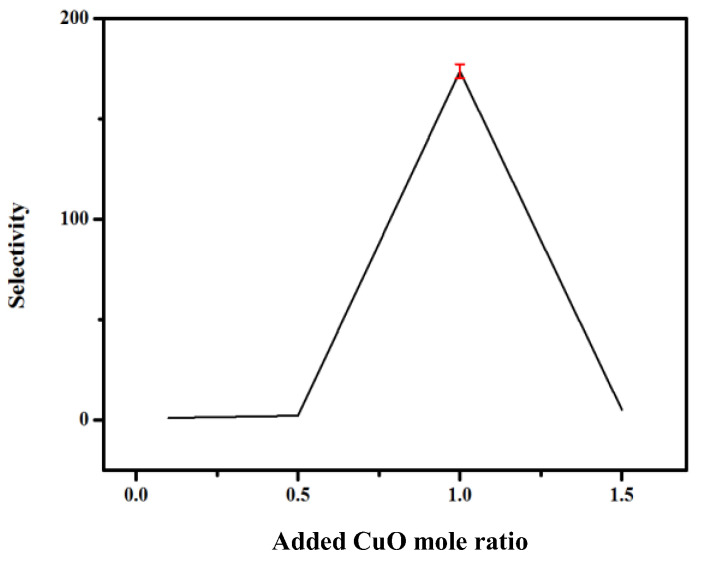
CO_2_/N_2_ separation performance of PVP/CuO/TCNQ membrane according to CuO.

**Figure 9:**
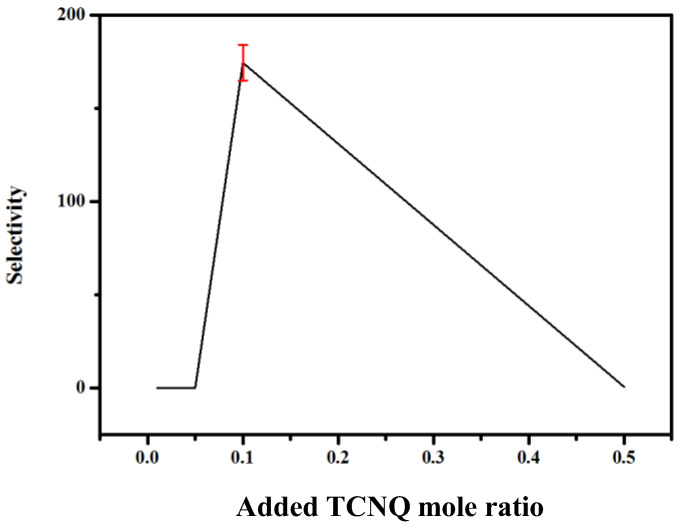
CO_2_/N_2_ separation performance of PVP/CuO/TCNQ membrane according to TCNQ.

The permeance of PVP/CuO/TCNQ film to CO_2_ and N_2_ gases was measured through a gas separation experiment. Basically, a single PVP film did not produce a significant difference in the degree of solubility for each gas when transmitting CO_2_ and N_2_ gases. Therefore, separation performance was not observed. Table 1 shows the separation performance of neat PVP, PVP/CuO composite, and PVP/CuO/TCNQ composite membranes. When only CuO was applied to PVP, gas permeance increased, but separation performance was not observed [28]. However, when TCNQ was added to the PVP/CuO composites, separation performance for CO_2_ was observed. The optimal ratio can be identified in Figure 8 and Figure 9, which show the change in selectivity according to the CuO and TCNQ contents. The selectivity was observed to be about 174.6, while the permeance of CO_2_ was 1.7 GPU. This shows that there was interaction between TCNQ and CuO, resulting in the enhancement in the separation performance by the modified surface property of particles.

**Scheme 1:**
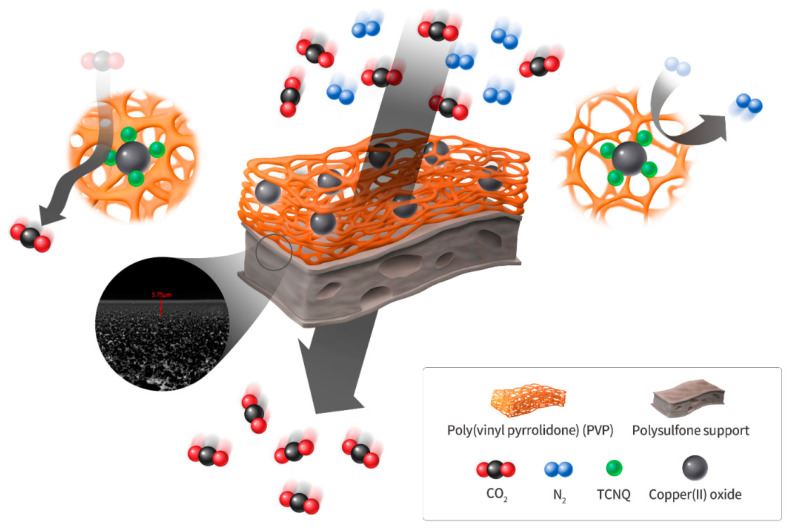
CO_2_ separation by PVP/CuO/TCNQ composite membrane.

In summary, the gas molecules could be transported by Fickian law to produce solution-diffusion transport. If solubility or diffusivity of specific molecules was increased by other factors, permeability increased. For the membranes utilized in this study, the solubility of CO_2_ molecules could be largely enhanced by the positively polarized surface of CuO particles as shown in Scheme 1.

## 3. Materials and Methods

### 3.1. Materials

Polyvinylpyrrolidone (PVP) (Mw≒360,000), 7,7,8,8-tetracyanoquinodimethane (TCNQ, 98%) and copper(II) oxide (CuO) were purchased from Aldrich Chemical Co. (St. Louis, MO, USA). Ethanol (94.5%) was purchased from DAEJUNG Chemicals & Metals Co. (Seoul, Republic of Korea). Commercial macroporous polysulfone was purchased from Toray Advanced Material Korea Inc. (Seoul, Republic of Korea).

### 3.2. Methods

#### 3.2.1. Membrane Preparation

The PVP/CuO/TCNQ composite membranes prepared based on polymer were prepared as follows: 3 wt % of polymer solution was prepared by dissolving PVP in ethanol. Thereafter, CuO and TCNQ were added at a ratio of 1/1/0.1 to PVP/CuO/TCNQ. Then, the mixture was stirred at 80 °C for 1 h. The prepared solution was applied as a coat on the macroporous polysulfone support membrane using a Model K202, Control Coater RK Print-Coat Instruments LTD (Litlington, UK). The coated membrane was dried in a thermostat oven for 1 h.

#### 3.2.2. Gas Permeance Experiments

The prepared membranes were measured immediately after drying at a constant temperature in a humidity chamber. The gas permeation experiment was conducted by permeating CO_2_ and N_2_ gases into the membrane. The pressure was 2 bar, and the permeance was measured using a bubble flow meter. A gas permeation unit (GPU) is 1 GPU = 1 × 10^−6^ cm^3^(STP)/(cm^2^ s cmHg).

#### 3.2.3. Characterization

The SEM images were observed using a JEOL JSM-5600LV (Akishima, Tokyo). Infrared data were measured using a VERTEX 70-FT IR spectrometer (Billerica, MA, USA). A total of 16 scans were signal-averaged with a resolution of 8 cm^−1^. The analysis was performed using thermogravimetric analysis (TGA Q50M, TA instruments, New Castle, DE, USA) to measure the thermal strength. UV was measured using a Genesys 10S UV–Vis spectrometer (ThermoFischer, Waltham, MA, USA).

## 4. Conclusions

In this study, CuO and TCNQ were added to PVP to create a polymer composite membrane. This was prepared by adding CuO and TCNQ to a PVP matrix. When CuO was added, the carbonyl bond was strengthened. Then, when TCNQ was also applied, the hydrogen bond between chains weakened, thereby relatively increasing the free volume. Furthermore, the particle size of CuO was small and well dispersed. The interaction between PVP, CuO, and TCNQ was analyzed through TGA, FT-IR, SEM, and UV–Vis. The results showed a CO_2_ permeance of 1.7 GPU and a CO_2_/N_2_ selectivity of 174. The solubility of CO_2_ increased due to the modified surface of CuO by TCNQ, and it was concluded that TCNQ improved the dispersion by preventing the agglomeration of CuO and positively polarized the surface, resulting in the increase in reversible interaction.

## Figures and Tables

**Table 1:** Separation performance of neat PVP, PVP/CuO, and PVP/CuO/TCNQ composite membranes.

	CO_2_/N_2_ Selectivity	CO_2_ Permeance (GPU)
Neat PVP	Not measurable	Not measurable
PVP/CuO	0.9	13.3
PVP/CuO/TCNQ	174 ± 9.5	1.7 ± 0.1

## Data Availability

Not applicable.
